# Knowledge, Attitudes and Practices of Pre‐Pregnancy Risk Management Among Childbearing‐Age Women With a Kidney Transplant: A Cross‐Sectional Study

**DOI:** 10.1002/nop2.70660

**Published:** 2026-07-12

**Authors:** Hangxia Ma, Weitong Lin, Meirong Wu, Jingjing Wan, Ke Cheng, Maosen Hu

**Affiliations:** ^1^ Nursing Department The Third Xiangya Hospital of Central South University Changsha Hunan China; ^2^ Nursing Department The First Affiliated Hospital of Zhengzhou University Zhengzhou Hunan China; ^3^ Xiangya College of nursing Central South University Changsha Hunan China; ^4^ Outpatient and Emergency Operating Room The Third Xiangya Hospital of Central South University Changsha Hunan China; ^5^ Department of Transplantation The Third Xiangya Hospital of Central South University Changsha Hunan China

**Keywords:** kidney transplantation, knowledge, attitude and practices (KAP), pre‐pregnancy risk management

## Abstract

**Background:**

Some women of child‐bearing age with a kidney transplant have a strong fertility desire, but they face a significantly higher risk of pregnancy than healthy women. Pre‐pregnancy risk management can help patients achieve planned pregnancies and reduce the risk factors for adverse pregnancy outcomes. Therefore, knowledge, attitudes, and practices (KAP) about pre‐pregnancy risk management are clinically important.

**Aims:**

To explore the current status and influencing factors of the KAP status of pre‐pregnancy risk management among Chinese women of child‐bearing age with a kidney transplant.

**Design:**

A cross‐sectional study.

**Methods:**

This cross‐sectional study used convenience sampling to recruit 247 women of child‐bearing age with a kidney transplant from 5 hospitals in Hunan Province from May to October 2024, using a 37‐item questionnaire developed and validated by multidisciplinary experts.

**Results:**

The mean KAP questionnaire was 135.04 ± 21.60, and the score rate was 72.99%. The scoring rates of knowledge, attitude and practices are 64.63%, 89.77% and 78.50%, respectively. Multiple linear regression analysis identified age, marital status, occupation, permanent residence and length of time after kidney transplantation as independent factors associated with pre‐pregnancy risk management KAP levels (*p* < 0.05).

**Conclusions:**

Women of child‐bearing age with a kidney transplant showed generally positive attitudes towards pre‐pregnancy risk management. However, their knowledge and practice levels need improvement. Healthcare providers should provide targeted health education to help them reduce pregnancy‐related risks.

**Impact:**

It is anticipated that this study will help nursing staff identify specific areas of inadequate knowledge for kidney transplant recipients before pregnancy and provide targeted health education to guide patients to reduce adverse outcomes for both mother and baby.

## 1 Introduction

Kidney transplantation is one of the most effective treatments for end‐stage renal disease. With the recovery of renal function, most women of child‐bearing age with a kidney transplant can regain normal sex hormone levels and fertility approximately 6 months after surgery (Klein and Josephson 2022; Katz‐Greenberg and Shah 2022). At present, women in the child‐bearing period and underage girls account for more than one‐sixth (approximately 16.7%) of kidney transplant recipients (Zhou et al. 2022; Wang and Zhou 2020). Some women of child‐bearing age with a kidney transplant have a strong reproductive need (Stavart et al. 2023), and successful childbearing is an important indicator of improved quality of life (Liu et al. 2018). However, they have higher pregnancy risks than healthy women and pregnancy may have various adverse effects on the transplant, maternal health and offspring outcome (Tangren et al. 2023). Pre‐pregnancy risk management can help patients achieve planned pregnancy, reduce the risk factors causing adverse pregnancy outcomes and improves pregnancy safety (Gosselink et al. 2022; Meinderts, Prins, et al. 2022; Meinderts, Schreuder, and de Schreuder 2022; Du 2020). According to the theoretical model of Knowledge, attitude and practices (KAP), female kidney transplant recipients who have a adequate knowledge and positive attitudes towards pre‐pregnancy risk management are more likely to implement relevant preventive behaviours and reduce pregnancy risks. However, research remains limited on KAP regarding pre‐pregnancy risk management or pre‐pregnancy health care among female kidney transplant recipients.

Given this, the study enrolled women of child‐bearing age with a kidney transplant to investigate their KAP status regarding pre‐pregnancy risk management. The findings aim to provide evidence for healthcare providers to strengthen health education and interventions and promote patients' understanding and practice of pre‐pregnancy risk management.

## 2 Methods

### 2.1 Study Design

A cross‐sectional design was adopted using convenience sampling. From May to October 2024, women of child‐bearing age with a kidney transplant attending outpatient departments or participating in WeChat follow‐up groups of five tertiary Grade A general hospitals in Hunan Province were invited to complete an electronic questionnaire via the Wenjuanxing platform (http://www.wjx.cn).

### 2.2 Participants

The inclusion criteria for the patients were as follows: ①History of at least one kidney transplant; ②Women aged between 15 and 49; ③Informed consent and voluntary participation in this study.

Patients with one or more of the following items were excluded: ① Patients with menopause or infertility such as sterilization and hysterectomy; ② Patients with severe mental illness or cognitive impairment who do not understand the meaning of the questionnaire.

### 2.3 Questionnaire Design

The questionnaire was developed by the research team under the guidance of a multidisciplinary expert group including transplant physicians, transplant nurses, obstetricians and gynaecologists. The process included literature review, semi‐structured expert and patient interviews, two rounds of expert consultation and a pilot survey.

The questionnaire demonstrated excellent reliability and validity: Cronbach's α coefficient for the total scale was 0.936. The Cronbach's α coefficient of the three parts of the questionnaire ranged from 0.705 to 0.945. The test–retest reliability of the total questionnaire was 0.883. The Scale‐Level Content Validity Index (S‐CVI) of the KAP questionnaire was 0.980, and the Item‐Level Content Validity Index (I‐CVI) coefficient for each item was between 0.87 and 1. The KMO values for knowledge, attitude and practice were 0.936, 0.917 and 0.666, respectively (all *p* < 0.01), meeting the criteria for factor analysis (KMO > 0.6, *p* < 0.05). Based on the scree plot (eigenvalue > 1) and cumulative variance (> 60%), four factors were extracted for the knowledge section (cumulative variance = 66.412%), one factor for the attitude section (cumulative variance = 62.094%, item loadings: 0.517–0.868) and two factors for the practice section (cumulative variance = 62.578%). All items had a good load (all > 0.4). After reliability and validity testing, the final formal 37‐item KAP questionnaire for pre‐pregnancy risk management among women of child‐bearing age with a kidney transplant was developed. See Data S1 for details.

The questionnaire contains two parts. The first part of the questionnaire collected demographic and disease information including age, marital status, fertility, occupation, education level, permanent residence, household income, payment method of medical expenses, length of time after kidney transplantation, dialysis duration and method, source of transplanted kidney, history of poor pregnancy outcomes, donation type and fertility desire. The second part was the KAP questionnaire, which included knowledge, attitude and practices, with a total of 37 entries. Knowledge part: There are 22 entries, including four dimensions, specifically for pregnancy risk decision (K1–K5), timing of pregnancy (K6–K10), medication and contraception during planned pregnancy (K11–K18), and routine self‐management (K19–K22). Attitudes part: a total of nine items, one dimension (A1–A9); Practices part: There are six items, including two dimensions, specifically knowledge reserve (P1, P2, P4) and behaviour management (P3, P6, P7). The Likert Grade 5 scoring method is adopted for these items.

### 2.4 Data Collection

The questionnaire was distributed by the investigators through the WeChat group of female kidney transplant recipients and out‐patient clinics of the organ transplantation department from May to October 2024, which ensured that the questionnaire was filled out for women after kidney transplantation. Quality control measures were applied: questionnaires completed in fewer than 4 min were deemed invalid; questionnaires with missing items could not be submitted; each WeChat account and IP address was restricted to one response to avoid duplicate submissions.

### 2.5 Statistical Analyses

Statistical analysis was performed using SPSS26.0 with a two‐sided test level of α = 0.05. Data were summarized using frequencies, percentages, means and standard deviations. For the difference test, independent sample *t‐*test and one‐way analysis of variance were used when the demographic and disease information met the normal distribution and the homogeneity of variance. Independent‐samples approximation *t*‐test and Kruskal–Wallis H test were used for uneven variances, and Bonferroni test was used for pairwise comparison between groups. Multivariate linear stepwise regression analysis was used for multivariate analysis of KAP scores.

### 2.6 Ethical Considerations

This study was conducted in strict accordance with the Declaration of Helsinki and approved by the Ethics Review Committee of Xiangya Nursing School of Central South University (No. E202112). Before the start of the investigation, all the investigated subjects were required to adopt a unified instruction to conduct a detailed description of the research, informing them of the purpose of the research and the time required for the research to obtain their informed consent. The questionnaire was filled out anonymously.

## 3 Results

### 3.1 Socio‐Demographic Characteristics

A total of 260 questionnaires were distributed, and 247 valid questionnaires were collected, with an effective recovery rate of 95.00%. The mean age of the respondents is 34.83 ± 6.54 years old, 70.45% of the women are married, 46.96% of them had no children at present and 42.91% of them have a clear desire to give birth. Other baseline characteristics are shown in Table 1.

**TABLE 1:** Sociodemographic information of the participants and single‐factor analysis of the factors influencing KAP (*n* = 247).

Item	Number (%)	Knowledge (mean ± SD)	Attitude (mean ± SD)	Practice (mean ± SD)	Total (mean ± SD)	Bonferroni
Age(years)						③ > ①*; ② > ⑤*; ③ > ④*; ③ > ⑤*
18~24①	13 (5.26)	65.92 ± 13.537	35.85 ± 8.821	21.85 ± 4.18	123.62 ± 20.148	
25~29②	30 (12.15)	76.87 ± 15.919	40.97 ± 4.93	24.93 ± 2.449	142.77 ± 19.618	
30~34③	95 (38.46)	75.66 ± 17.24	41.28 ± 4.239	24.26 ± 3.183	141.21 ± 20.103	
35~39④	62 (25.10)	67.5 ± 17.62	39.76 ± 6.642	23.63 ± 3.46	130.89 ± 22.366	
40~49⑤	47 (19.03)	64.34 ± 15.341	40.36 ± 5.708	21.57 ± 4.451	126.28 ± 20.009	
t/F value		5.645^a^	6.230^c^	19.834^c^	6.925^a^	
*p*‐value		0.000*	0.183	0.001*	0.000*	
Marital status						① > ④*; ② > ④*
Unmarried①	50 (20.24)	69.96 ± 16.25	40.92 ± 5.45	23.80 ± 3.25	134.68 ± 19.35	
Married②	174 (70.45)	72.68 ± 17.23	40.25 ± 5.57	23.59 ± 3.81	136.51 ± 21.93	
Divorce③	21 (8.50)	62.90 ± 17.60	42.05 ± 4.52	23.05 ± 3.34	128.00 ± 19.48	
Widowed④	2 (0.81)	47.50 ± 4.95	23.50 ± 4.95	19.00 ± 2.83	90.00 ± 12.73	
t/F value		3.473^a^	7.214^a^	1.246^a^	4.064^a^	
*p‐*value		0.017*	0.000*	0.294	0.008*	
Number of pregnancies						① > ③*
0①	86 (34.82)	72.59 ± 16.53	40.88 ± 4.869	24.28 ± 3.497	137.76 ± 19.628	
1②	74 (29.96)	75.65 ± 17.626	40.03 ± 6.762	23.53 ± 3.854	139.20 ± 24.015	
2③	43 (17.41)	66.28 ± 17.126	39.88 ± 5.708	23.35 ± 2.919	129.51 ± 21.246	
≥ 3④	44 (17.81)	65.2 ± 16.033	40.59 ± 5.132	22.34 ± 4.069	128.14 ± 19.047	
t/F value		4.971^a^	0.448^a^	2.836^a^	3.944^a^	
*p‐*value		0.002*	0.719	0.039*	0.009*	
Number of children						
0①	116 (46.96)	73.37 ± 16.609	40.74 ± 5.123	24.40 ± 3.233	138.51 ± 20.459	
1②	105 (42.51)	68.95 ± 17.786	40.5 ± 6.216	22.78 ± 3.805	132.23 ± 22.259	
2③	19 (7.69)	71.95 ± 17.312	39.21 ± 5.04	24.26 ± 3.124	135.42 ± 20.006	
More than 2	7 (2.83)	63.14 ± 18.106	36.57 ± 6.579	19.00 ± 4.509	118.71 ± 25.085	
t/F value		1.732^a^	1.504^a^	8.08^a^	2.997^a^	
*p‐*value		0.161	0.214	0.000*	0.031*	
Occupation						① > ②*
In service①	138 (55.87)	74.06 ± 16.846	40.93 ± 4.89	24.08 ± 3.379	139.07 ± 20.433	
Others②	109 (44.13)	67.34 ± 17.186	39.72 ± 6.478	22.87 ± 3.911	129.94 ± 22.038	
t/F value		3.085^b^	1.673^b^	2.602^b^	3.37^b^	
*p‐*value		0.002*	0.107	0.010*	0.000*	
Education level						
Junior high school and below	30 (12.15)	66.70 ± 16.434	40.33 ± 5.962	22.2 ± 4.723	129.23 ± 21.161	
High school/ technical secondary	77 (31.17)	67.96 ± 16.809	39.52 ± 5.44	23.61 ± 3.671	131.09 ± 21.362	
junior college	63 (25.51)	73.52 ± 16.204	41.25 ± 5.688	23.67 ± 3.436	138.44 ± 20.084	
Undergraduate	66 (26.72)	73.15 ± 18.42	40.88 ± 5.859	23.62 ± 3.322	137.65 ± 22.648	
Master or above	11 (4.45)	78.73 ± 18.056	39 ± 4.879	25.64 ± 2.838	143.36 ± 21.369	
t/F value		2.242^a^	1.111^a^	4.750^c^	2.273^a^	
*p‐*value		0.065	0.352	0.314	0.062	
Permanent residence						③ > ①*; ③ > ②*
Rural①	54 (21.86)	68.81 ± 16.114	39.59 ± 6.784	23.37 ± 4.297	131.78 ± 22.182	
County town②	69 (27.94)	66.45 ± 16.398	38.74 ± 6.818	22.17 ± 3.502	127.36 ± 20.672	
Urban③	124 (50.20)	74.67 ± 17.606	41.68 ± 3.898	24.39 ± 3.218	140.73 ± 20.35	
t/F value		5.831^a^	7.313^c^	8.669^a^	9.963^a^	
*p‐*value		0.003*	0.026*	0.000*	0.000*	
Household income (RMB)						④ > ①*
Within 3000①	58 (23.48)	65.59 ± 16.515	39.48 ± 6.762	22.69 ± 4.092	127.76 ± 21.165	
3001 ~ 5000②	101 (40.89)	71.94 ± 17.521	40.53 ± 5.642	23.16 ± 3.719	135.63 ± 22.318	
5001 ~ 8000③	49 (19.84)	73.22 ± 16.413	40.63 ± 5.43	24.53 ± 3.386	138.39 ± 20.859	
More than 8000④	39 (15.79)	74.41 ± 17.674	41.13 ± 4.034	24.59 ± 2.663	140.13 ± 19.082	
t/F value		2.831^a^	0.766^a^	3.789^a^	3.436^a^	
*p‐*value		0.039*	0.514	0.011*	0.018*	
Payment method of medical expenses						
Employees medical insurance	127 (51.42)	73.11 ± 17.326	41.07 ± 4.643	23.93 ± 3.654	138.11 ± 20.992	
Urban medical insurance	52 (21.05)	71.65 ± 17.383	39.25 ± 6.747	22.87 ± 3.581	133.77 ± 21.058	
New rural cooperative medical insurance	64 (25.91)	66.47 ± 16.939	40.13 ± 6.48	23.27 ± 3.679	129.86 ± 22.75	
Pay one's own expenses	4 (1.62)	73.75 ± 9.811	38.50 ± 4.796	24.75 ± 4.573	137 ± 18.019	
t/F value		2.183^a^	2.995^c^	1.334^a^	2.185^a^	
*p‐*value		0.091	0.392	0.264	0.09	
Length of time after kidney transplantation						④ > ①*; ④ > ③*
Within 1 year①	24 (9.72)	66.29 ± 14.956	36.83 ± 7.551	21.71 ± 4.704	124.83 ± 21.661	
1 ~ 2 years②	58 (23.48)	66.98 ± 16.888	41.4 ± 5.541	24.02 ± 2.953	132.40 ± 19.657	
3 ~ 5 years③	59 (23.89)	68.25 ± 17.158	39.58 ± 6.259	22.93 ± 3.643	130.76 ± 22.813	
6 ~ 10 years④	79 (31.98)	76.63 ± 15.921	41.48 ± 4.203	24.10 ± 3.643	142.22 ± 18.971	
More than 10 years⑤	27 (10.93)	74.19 ± 20.178	40.07 ± 5.144	23.89 ± 3.672	138.15 ± 24.299	
t/F value		4.123^a^	14.105^c^	8.662^a^	4.726^a^	
*p‐*value		0.003*	0.007*	0.07	0.001*	
Dialysis duration						
No dialysis	22 (8.91)	69.55 ± 17.706	37.64 ± 7.095	23.55 ± 3.569	130.73 ± 22.979	
Within 3 months	45 (18.22)	73.56 ± 19.227	40.53 ± 5.864	23.36 ± 3.961	137.44 ± 23.592	
3–6 months	42 (17.00)	71.07 ± 14.181	38.76 ± 6.339	23.07 ± 3.564	132.9 ± 19.187	
7–11 months	46 (18.62)	74.8 ± 15.998	40.85 ± 5.007	24.04 ± 4.016	139.7 ± 20.684	
1–3yeras	58 (23.48)	70.22 ± 18.712	41.84 ± 4.934	24.31 ± 3.004	136.38 ± 22.386	
More than 3 years	34 (13.77)	65.32 ± 16.352	40.97 ± 4.739	22.41 ± 3.839	128.71 ± 19.789	
t/F value		1.441^a^	2.727^a^	1.505^a^	1.439^a^	
*p‐*value		0.21	0.020*	0.189	0.211	
Preoperative dialysis method						
Haemodialysis	171 (69.23)	71.64 ± 17.236	40.88 ± 5.272	23.51 ± 3.884	136.04 ± 21.642	
Peritoneal dialysis	45 (18.22)	69.73 ± 17.432	40.2 ± 6.628	23.33 ± 3.536	133.27 ± 21.937	
Haemodialysis and Peritoneal dialysis	20 (8.10)	70.75 ± 18.307	38.35 ± 5.715	23.85 ± 2.455	132.95 ± 22.277	
No dialysis	11 (4.45)	68.73 ± 17.568	37.55 ± 6.362	24.36 ± 2.541	130.64 ± 19.735	
t/F value		0.22^a^	2.261^a^	7.518^c^	0.434^a^	
*p‐*value		0.883	0.082	0.185	0.729	
History of bad maternity						
Spontaneous abortion	24 (9.72)	68.88 ± 14.468	38.17 ± 6.939	23.29 ± 3.407	130.33 ± 20.067	
Induced abortion	75 (30.36)	67.92 ± 17.783	41.13 ± 4.548	23.56 ± 3.146	132.61 ± 20.525	
No	148 (59.92)	73.06 ± 17.274	40.39 ± 5.89	23.58 ± 3.959	137.03 ± 22.245	
t/F value		2.448^a^	3.330^c^	0.065^a^	1.683^a^	
*p‐*value		0.089	0.189	0.937	0.188	
Donation type						
Donation after death	193 (78.14)	70.13 ± 17.498	40.67 ± 5.434	23.42 ± 3.625	134.22 ± 21.505	
Living donation	54 (21.86)	74.54 ± 16.207	39.43 ± 6.389	24.00 ± 3.807	137.96 ± 21.87	
t/F value		−1.662^b^	1.433^b^	−1.029^b^	−1.126^b^	
*p‐*value		0.098	0.153	0.305	0.261	
Fertility desire						① > ②*; ① > ③*
Yes①	106 (42.91)	77.42 ± 16.458	40.29 ± 5.397	24.85 ± 3.144	142.57 ± 20.754	
Not sure②	52 (21.05)	67.52 ± 14.075	39.29 ± 5.326	23.31 ± 3.317	130.12 ± 16.887	
No③	89 (36.03)	65.64 ± 17.653	41.18 ± 6.099	22.13 ± 3.911	128.96 ± 22.464	
t/F value		13.981^a^	1.876^a^	14.920^a^	12.367^a^	
*p‐*value		0.000*	0.155	0.000*	0.000*	

* Indicated *p* < 0.05.

^a^ One‐way analysis of variance, post hoc comparisons were performed using the Bonferroni correction.

^b^ Two‐independent sample *t*‐test.

^c^ Kruskal–Wallis H test.

### 3.2 KAP Scores of Pre‐Pregnancy Risk Management in Women of Child‐Bearing Age With a Kidney Transplant

The total score of the KAP questionnaire was 185. The mean score was 135.04 ± 21.60, with a score rate of 72.99%. The score rates for knowledge, attitude and practice were 64.63%, 89.77% and 78.50%, respectively. Among the four dimensions of knowledge, the highest score rate was observed for pregnancy timing, and the lowest for pre‐pregnancy medication and contraception (Table 2). In addition, the knowledge item with the highest score was “I know it is not suitable to be pregnant if rejection has occurred recently”, and the knowledge item with the lowest scores was “I know that certain antihypertensive medications, such as captopril, enalapril, losartan and valsartan, may cause foetal malformations during pregnancy”. The item with the highest attitude score was “I am willing to prevent infection and monitor my condition under the guidance of medical staff”, and the item with the lowest score was “If the financial situation and the support of family members are poor, I am willing to make a more cautious decision about whether to have children”. Among the two dimensions of practices, the knowledge reserve dimension was used to investigate the behaviour of women of child‐bearing age with a kidney transplant in acquiring relevant knowledge, whereas the practices dimension was used to investigate the degree of self‐management of related behaviours in women of child‐bearing age with a kidney transplant. Scores for the knowledge reserve dimension are lower than those for the practices dimension. Of the two dimensions of practices, the item with the highest score was “I can strictly adhere to medical advice and schedule regular disease follow‐ups”, and the item with the lowest score was “I can actively consult a doctor on fertility‐related issues”.

**TABLE 2:** Knowledge, Attitude and Practice (KAP) mean scores and accuracy rates of female kidney transplant recipients on pre‐pregnancy risk management (*n* = 247).

Category	Dimension	Item	Highest score	Lowest score	Mean score for each dimension (*x* ± *s*)	Score rate (%)
Knowledge		22	105	22	71.09 ± 17.29	64.63
	Pregnancy risk decisions	5	25	5	15.95 ± 4.21	63.80
	Timing of pregnancy	5	25	5	17.29 ± 4.80	69.16
	Medication and contraception during planned pregnancy	8	40	8	24.45 ± 7.02	61.13
	Routine self management	4	20	4	13.41 ± 3.58	67.05
Attitude		9	45	15	40.40 ± 5.67	89.77
Practice		6	30	15	23.55 ± 3.67	78.50
	Knowledge reserve	3	15	3	10.86 ± 2.69	72.40
	Behaviour management	3	15	4	12.67 ± 1.99	84.47
Total		37	177	62	135.04 ± 21.60	72.99

### 3.3 Univariate Analysis of Influencing KAP About Pre‐Pregnancy Risk Management in Women of Child‐Bearing Age With a Kidney Transplant

Univariate analysis revealed significant differences in KAP total scores by age, marital status, number of pregnancies, number of children, occupation, permanent residence, household income, length of time after kidney transplantation, and fertility desire (*p* < 0.05) (Table 1).

### 3.4 Multivariate Analysis of Influencing KAP About Pre‐Pregnancy Risk Management in Women of Child‐Bearing Age With a Kidney Transplant

The factors influencing the total scores of KAP were taken as independent variables in the results of single factor analysis, and the total scores of KAP were taken as dependent variables for multivariate linear regression analysis. The influencing factors of the KAP total score of pre‐pregnancy risk management for women of child‐bearing age with a kidney transplant were age, marital status, occupation, permanent residence and length of time after kidney transplantation. The independent variable assignment methods and the results of the analysis are shown in Table 3 and Table 4, respectively.

**TABLE 3:** Independent variable assignment.

Independent variable	Assignment method
Age	18~24 = 1; 25~29 = 2; 30~34 = 3; 35~39 = 4; 40~49 = 5
Marital status	Set dummy variable with married as reference, married (Z_1_ = 0, Z_2_ = 0, Z_3_ = 0); unmarried (Z_1_ = 1, Z_2_ = 0, Z_3_ = 0); divorce (Z_1_ = 0, Z_2_ = 1, Z_3_ = 0); widowed (Z_1_ = 0, Z_2_ = 0, Z_3_ = 1)
Number of children	0 = 1; 1 = 2; 2 = 3; More than 2 = 4
Occupation	In service = 1; others = 2
Permanent residence	Set dummy variable with county town as reference, county town (Z_1_ = 0, Z_2_ = 0); rural (Z_1_ = 1, Z_2_ = 0); Urban (Z_1_ = 0, Z_2_ = 1)
Household income (RMB)	Within 3000 = 1; 3001~5000 = 2; 5001~8000 = 3; More than 8000 = 4
Length of time after kidney transplantation	Within 1 year = 1; 1~2 years = 2; 3~5 years = 3; 6~10 years = 4; More than 10 years = 5
Dialysis duration	No dialysis = 1; Within 3 months = 2; 3~6 months; 7~11 months = 4; 1–3 years = 5; More than 3 years = 6
Fertility desire	Yes = 1; Not sure = 2; No = 3

**TABLE 4:** Multivariate linear stepwise regression analysis of factors influencing the total score of knowledge, attitude and practices.

Independent variable	Non‐standardized regression coefficient	Standard error	Standardized regression coefficient	*t*	*p*
Constant	125.569	8.390	—	14.966	0.000
Age	−2.976	1.409	−0.15	−2.112	0.036
Marital status					
Unmarried	−7.715	3.793	−0.144	−2.034	0.043
Widowed	−42.83	14.008	−0.178	−3.058	0.002
Occupation	5.980	2.783	0.138	2.149	0.033
Permanent residenc (Urban)	11.026	3.031	0.256	3.638	0.000
Length of time after kidney transplantation	3.436	1.121	0.187	3.065	0.002

*Note:* KAP: *R* ^2^ = 0.242, adjusted *R* ^2^ = 0.203; *F* = 6.225.

## 4 Discussion

In this study, the score rates of total KAP, knowledge, attitude and practice were 72.99%, 64.63%, 89.77% and 78.50%, respectively. According to the calculation formula (Perneger et al. 2015): scoring rate = (actual score of a project/the highest possible score of the project) × 100%. Previous studies classified score rates < 10% as low, 60%–79% as moderate and ≥ 80% as high (Jv et al. 2020). So the total score, knowledge score and practices score were at a moderate level, while attitude scores were at a high level. The attitude to pre‐pregnancy risk management of reproductive female kidney transplant recipients was relatively positive; however, the level of knowledge and practice required improvement.

Among the four knowledge dimensions, the lowest score rate was found for pre‐pregnancy medication and contraception. The three lowest scoring knowledge items were as follows: first, “I know that certain antihypertensive medications, such as captopril, enalapril, losartan and valsartan, may cause foetal malformations during pregnancy”; second, “I know that it is possible and relatively safe for women with functioning kidney transplants to have children” and third, “I know that oestrogen‐containing contraceptives may increase the risk of blood clots and aggravate high blood pressure. Women with vascular disease and high blood pressure should avoid using them”. These findings indicate insufficient knowledge among participants regarding three key domains: teratogenic risks of common antihypertensive medications, the relative safety of pregnancy following kidney transplantation, and contraceptive‐related risks and recommendations. Blood pressure control before and during pregnancy is critical, with a recommended pre‐pregnancy target of < 140/90 mmHg (Webster et al. 2019). The angiotensin‐converting enzyme inhibitor and angiotensin receptor antagonist may cause foetal growth restriction and deformity, and they should be stopped before pregnancy (Stavart et al. 2023). Consistent with our results, a previous KAP study on pre‐pregnancy care similarly identified medication‐related teratogenic risks as the least understood knowledge component among women of childbearing age. Evidence further confirms that pregnancy does not directly impair long‐term renal allograft function, with graft loss rates comparable to those of the general transplant recipient population, suggesting acceptable pregnancy‐related risks for kidney transplant recipients (Kaatz et al. 2023; Schwarz et al. 2022). The study (French et al. 2013) shows that 44% of female transplant recipients were unaware of post‐transplant pregnancy possibility, and only 36% received pre‐transplant contraceptive education from health‐care providers. Additional studies have similarly documented low rates of post‐transplant contraceptive counselling among recipients; receipt of such counselling significantly predicts effective contraceptive use (Ritchie et al. 2020; Szpotanska‐Sikorska et al. 2014).

Among the attitude items, the item with the lowest score was “If the financial situation and the support of family members are poor, I am willing to make a more cautious decision about whether to have children”. Studies have shown that family support and financial and material support for pregnant women can improve the compliance of pregnant women with treatment, and improve their mental health and pregnancy outcome (Pereira et al. 2023). One participant with a history of preterm birth in the qualitative interviews reported that the main reasons for her adverse pregnancy outcomes were poor financial conditions and lack of support from her family, which led to severe negative emotions during pregnancy. Therefore, medical staff should emphasize the importance of family support and financial readiness to help patients make informed pregnancy decisions.

The entry with the lowest score was “I can actively consult a doctor on fertility‐related issues”, suggesting that proactive help‐seeking behaviour for fertility counselling requires improvement. Studies have shown that few women who have undergone transplantation have received health education on the effects of organ transplantation on fertility (Oki et al. 2023). Therefore, it is suggested that medical staff should include fertility counselling as part of routine care for women of child‐bearing age with a kidney transplant, and at the same time, women who desire a family should be encouraged to take the initiative in fertility counselling.

Multivariate analysis confirmed that age, marital status, occupation, permanent residence and length of time after kidney transplantation. Age was inversely proportional to the total scores of KAP and Knowledge. It may be that older people are less able to actively learn and accept things. Studies have demonstrated that cognitive function and memory capacity decline progressively with age. Older individuals tend to experience impaired cognition and reduced learning competence, making it harder for them to effectively acquire and grasp new knowledge compared with younger counterparts (Song et al. 2024). A similar trend was reported in a study on reproductive health KAP, where middle‐aged participants showed lower reproductive health knowledge (Li 2023). Married women scored higher than others, which is consistent with married women's reproductive needs. Unmarried and widowed people may consider pregnancy a low priority. Working women can get economic income, participate in more social activities and get information about safe pregnancy more easily. A study showed that the quality of life and social support of employed kidney transplant recipients after operation were higher than those of unemployed kidney transplant recipients (Rao et al. 2023). It is more convenient for people living in cities to seek medical treatment and obtain more forms of health education, and they have a stronger awareness of paying attention to pre‐pregnancy health care and safe pregnancy. Physician communication strongly influences pregnancy‐related knowledge among transplant recipients (Tong et al. 2015). Longer post‐transplantation duration was associated with higher KAP scores. This may be due to the fact that the primary goal of early kidney transplantation patients is to maintain the stability of the graft rather than pregnancy. Wang also showed that the longer the time after kidney transplantation, the higher the quality of life and health literacy (Wang et al. 2019). With the gradual increase of postoperative time, the function of the graft becomes more stable and the physical condition is easier to achieve the basic conditions of pregnancy. Therefore, the study of relevant knowledge has increased and the attitude is more positive.

The present findings provide practical and actionable guidance for transplant nurses in the reproductive health management of childbearing‐age women with a kidney transplant. First, nurses are able to identify high‐risk patients with inadequate pre‐pregnancy knowledge and deliver targeted one‐on‐one education focusing on teratogenic antihypertensive agents and safe contraceptive strategies. Second, fertility screening should be integrated into routine outpatient follow‐up, with proactive pre‐pregnancy risk counselling delivered prior to patients' conception plans. For recipients with complicated physical conditions or financial constraints, nurses can facilitate multidisciplinary referrals to transplant specialists and obstetricians to optimise pre‐conception preparation. Furthermore, regular follow‐up is essential to encourage patients to actively seek professional medical advice regarding fertility issues, rather than relying on passive clinical guidance.

### 4.1 Limitations

This study has some limitations. First, the survey was only conducted in a few hospitals in Hunan Province rather than nationwide, which has certain geographical limitations. Second, the cross‐sectional design prevents evaluation of changes in the KAP level over time. In addition, the convenience sampling method conducted through WeChat and outpatient clinics has the risk of selection bias, which may have a certain impact on the representativeness and generalizability of the sample.

## 5 Conclusion

In conclusion, our results show that women of child‐bearing age with a kidney transplant show moderate KAP levels regarding pre‐pregnancy risk management, with positive attitudes but deficiencies in knowledge and practice. Independent influencing factors include age, marital status, occupation, permanent residence and length of time after kidney transplantation. Therefore, medical staff should develop individualized health education strategies to improve pre‐pregnancy knowledge and self‐management behaviours in this high‐risk population.

## Author Contributions

H.M. and W.L. developed the study idea and design, collected data, conducted data analysis and drafted the manuscript. M.H. helped collect data, conducted data analysis and searched literature. M.W. contributed to the manuscript revision. J.W. and K.C. provided critical feedback on the manuscript and helped with the study design and manuscript revision. All authors critically reviewed the manuscript and approved the final version.

## Funding

This work was supported by the Hunan Provincial Natural Science Foundation General Project (2023JJ30844).

## Disclosure

No AI writing assistance was used in this study.

## Conflicts of Interest

The authors declare no conflicts of interest.

## Supporting information


**Data S1:** Knowledge, attitude and practice questionnaire on pre‐pregnancy risk management among childbearing‐age women with a kidney transplant.

## Data Availability

The data that support the findings of this study are available from the corresponding author upon reasonable request.
