# Supplementary material for: Knowledge, Attitudes and Practices of Pre‐Pregnancy Risk Management Among Childbearing‐Age Women With a Kidney Transplant: A Cross‐Sectional Study
Source: Nurs Open. 2026 Jul 12;13(7):e70660. doi: 10.1002/nop2.70660 (PMC13357685; doi:10.1002/nop2.70660)
Supplement: Supplementary file 1 — Data S1: Knowledge, attitude and practice questionnaire on pre‐pregnancy risk management among childbearing‐age women with a kidney transplant. [file NOP2-13-e70660-s001.docx]

**Knowledge, Attitude, and Practice Questionnaire on Pre-pregnancy Risk Management Among Childbearing-age Women With a Kidney Transplant**

The following are descriptions of knowledge, attitudes, and behaviors regarding pre-pregnancy risk management among childbearing-age women with a kidney transplant. Please check (✓) the appropriate box according to your understanding.

1. Necessary Knowledge for Pre-pregnancy Risk Management Among Childbearing-age Women With a Kidney Transplant

| **Knowledge Items** | **Strongly Disagree** | **Disagree** | **Uncertain** | **Agree** | **Strongly Agree** |
| --- | --- | --- | --- | --- | --- |
| K1. I know that women with normal transplant kidney function can have children, and it is relatively safe. |  |  |  |  |  |
| K2. I know that women after kidney transplantation have a higher risk of complications such as gestational hypertension, diabetes, preterm birth, and low birth weight infants than the general female population. |  |  |  |  |  |
| K3. I know that pre-pregnancy creatinine, proteinuria, and hypertension increase the risk of deterioration or even loss of transplant kidney function. |  |  |  |  |  |
| K4. I know that women over 35 years old (advanced maternal age), obese, malnourished, or with a history of adverse pregnancy outcomes have a higher risk of pregnancy complications. |  |  |  |  |  |
| K5. I know that if I want to become pregnant, I should consult a doctor to see if I am suitable for pregnancy and jointly formulate a pregnancy preparation plan with the doctor. |  |  |  |  |  |
| K6. I know that it is suitable to become pregnant at least 1 year after kidney transplantation with good overall health status. |  |  |  |  |  |
| K7. I know that pregnancy is not suitable if rejection has occurred recently. |  |  |  |  |  |
| K8. I know that before pregnancy, efforts should be made to achieve stable transplant function (creatinine ≤133 µmol/L), normal blood pressure, and no proteinuria. |  |  |  |  |  |
| K9. I know that pre-pregnancy renal ultrasound examination should be performed to rule out hydronephrosis, renal calculi, etc., in the transplanted kidney before pregnancy is suitable. |  |  |  |  |  |
| K10. I know that pre-pregnancy prenatal health examinations should be performed 3-6 months before pregnancy, and pregnancy should be delayed until abnormalities are treated and normalized. |  |  |  |  |  |
| K11. I know that some immunosuppressants may need to be reduced or replaced during pregnancy. |  |  |  |  |  |
| K12. I know that the use of certain immunosuppressants before and during pregnancy, such as mycophenolate mofetil, sirolimus, and everolimus, may cause miscarriage or fetal malformation. |  |  |  |  |  |
| K13. I know that pregnancy can only be attempted at least 6 weeks after discontinuing teratogenic immunosuppressants. |  |  |  |  |  |
| K14. I know that certain antihypertensive medications, such as captopril, enalapril, losartan, and valsartan, may cause fetal malformations during pregnancy. |  |  |  |  |  |
| K15. I know that strict contraception should be practiced until a suitable gestational timing is reached. |  |  |  |  |  |
| K16. I know that contraception using barrier methods or intrauterine devices is safer. |  |  |  |  |  |
| K17. I know that estrogen-containing contraceptives increase the risk of thrombosis and aggravate hypertension, and should be avoided by women with vascular diseases or hypertension. |  |  |  |  |  |
| K18. I know that emergency contraceptives can be taken when there is a risk of unintended pregnancy, regardless of the stability of transplant kidney function. |  |  |  |  |  |
| K19. I know that healthy lifestyle should be maintained before pregnancy, including regular work and rest, smoking cessation and alcohol avoidance, avoiding exposure to high-risk substances (e.g., radiation, heavy metals), avoiding close contact with pets, and maintaining mental health. |  |  |  |  |  |
| K20. I know that vitamins such as folic acid should be supplemented at least 1-3 months before pregnancy, nutrition should be balanced, and immune-enhancing foods such as fungus, mushrooms, honey, and ginseng should be avoided. |  |  |  |  |  |
| K21. I know that pre-pregnancy weight should be controlled through diet and exercise to achieve a suitable BMI (18.5-23.9 kg/m²). |  |  |  |  |  |
| K22. I know that taking medications as prescribed, monitoring blood pressure, observing the color and characteristics of urine (e.g., foamy urine) and urine output, and identifying symptoms of disease exacerbation are important self-management methods before pregnancy. |  |  |  |  |  |

1. Beliefs and Attitudes Regarding Pre-pregnancy Risk Management Among Childbearing-age Women With a Kidney Transplant

| **Attitude Items** | **Strongly Disagree** | **Disagree** | **Uncertain** | **Agree** | **Strongly Agree** |
| --- | --- | --- | --- | --- | --- |
| A1. If I want to become pregnant, I am willing to proactively consult doctors for relevant knowledge. |  |  |  |  |  |
| A2. If the financial situation and the support of family members are poor, I am willing to make a more cautious decision about whether to have children |  |  |  |  |  |
| A3. If I decide to have children, I am willing to undergo comprehensive pre-pregnancy examinations in transplant departments, obstetrics and gynecology departments, etc., as prescribed by doctors. |  |  |  |  |  |
| A4. If the suitable gestational timing has not been reached, I am willing to delay pregnancy as prescribed by doctors. |  |  |  |  |  |
| A5. If medication adjustment is needed before pregnancy, I am willing to adjust medication as prescribed by doctors. |  |  |  |  |  |
| A6. If medication adjustment may require more frequent follow-up examinations, I am willing to cooperate. |  |  |  |  |  |
| A7. If contraception is needed, I am willing to use safe and reliable contraception strictly according to the guidance of medical staff. |  |  |  |  |  |
| A8. If I have unhealthy lifestyles in terms of work and rest, diet, and exercise during pregnancy preparation, I am willing to change them. |  |  |  |  |  |
| A9. I am willing to prevent infections and monitor my condition under the guidance of medical staff. |  |  |  |  |  |

1. Behaviors Regarding Pre-pregnancy Risk Management Among Childbearing-age Women With a Kidney Transplant

| **Behavior Items** | **Strongly Disagree** | **Disagree** | **Uncertain** | **Agree** | **Strongly Agree** |
| --- | --- | --- | --- | --- | --- |
| P1. I actively understand and learn knowledge related to fertility after kidney transplantation. |  |  |  |  |  |
| P2. I can proactively consult doctors about fertility-related issues. |  |  |  |  |  |
| P3. I can strictly adhere to medical advice and schedule regular disease follow-ups |  |  |  |  |  |
| P4. I can become pregnant at the appropriate time according to doctors' guidance. |  |  |  |  |  |
| P5. I have never stopped or reduced medication on my own because I wanted to become pregnant. |  |  |  |  |  |
| P6. I do not use unreliable contraceptive methods such as rhythm method or withdrawal. |  |  |  |  |  |
